# Nephrotic syndrome‐associated hypercoagulopathy is alleviated by both pioglitazone and glucocorticoid which target two different nuclear receptors

**DOI:** 10.14814/phy2.14515

**Published:** 2020-08-09

**Authors:** Amanda P. Waller, Shipra Agrawal, Katelyn J. Wolfgang, Jiro Kino, Melinda A. Chanley, William E. Smoyer, Bryce A. Kerlin, the Pediatric Nephrology Research Consortium (PNRC), J Mahan, H Patel, RF Ransom, C Pan, DF Geary, ML Chang, KL Gibson, FM Iorember, PD Brophy, T Srivastava, LA Greenbaum

**Affiliations:** ^1^ Center for Clinical & Translational Research The Abigail Wexner Research Institute at Nationwide Children's Columbus OH USA; ^2^ Department of Pediatrics The Ohio State University College of Medicine Columbus OH USA

**Keywords:** Hypercoagulopathy, Methylprednisolone, Nephrotic Syndrome, Nuclear Receptors, Pioglitazone, Thrombosis

## Abstract

**Background:**

Thrombosis is a potentially life‐threatening nephrotic syndrome (NS) complication. We have previously demonstrated that hypercoagulopathy is proportional to NS severity in rat models and that pioglitazone (Pio) reduces proteinuria both independently and in combination with methylprednisolone (MP), a glucocorticoid (GC). However, the effect of these treatments on NS‐associated hypercoagulopathy remains unknown. We thus sought to determine the ability of Pio and GC to alleviate NS‐associated hypercoagulopathy.

**Methods:**

Puromycin aminonucleoside‐induced rat NS was treated with sham, Low‐ or High‐dose MP, Pio, or combination (Pio + Low‐MP) and plasma was collected at day 11. Plasma samples were collected from children with steroid‐sensitive NS (SSNS) and steroid‐resistant NS (SRNS) upon presentation and after 7 weeks of GC therapy. Plasma endogenous thrombin potential (ETP), antithrombin (AT) activity, and albumin (Alb) were measured using thrombin generation, amidolytic, and colorimetric assays, respectively.

**Results:**

In a rat model of NS, both High‐MP and Pio improved proteinuria and corrected hypoalbuminemia, ETP and AT activity (*p* < .05). Proteinuria (*p* = .005) and hypoalbuminemia (*p* < .001) were correlated with ETP. In childhood NS, while ETP was not different at presentation, GC therapy improved proteinuria, hypoalbuminemia, and ETP in children with SSNS (*p* < .001) but not SRNS (*p* = .330).

**Conclusions:**

Both Pio and GC diminish proteinuria and significantly alleviate hypercoagulopathy. Both Pio and MP improved hypercoagulopathy in rats, and successful GC therapy (SSNS) also improved hypercoagulopathy in childhood NS. These data suggest that even a partial reduction in proteinuria may reduce NS‐associated thrombotic risk.

## Graphical abstract

Acquired hypercoagulopathy contributes to thrombotic‐risk in patients with nephrotic syndrome. Here we show that treatments directed at reversing the glomerular filtration defect effectively reduce hypercoagulopathy in both animal models and childhood nephrosis. These data imply that effective treatment for nephrotic syndrome may simultaneously reduce thrombotic risk.

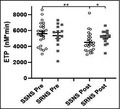

## 1 INTRODUCTION

Nephrotic syndrome (NS) is characterized by glomerular injury and massive urinary protein loss, which leads to a severe, acquired hypercoagulopathy associated with an elevated risk for life‐threatening venous thromboembolic (VTE) disease that afflicts up to 25% of adult and 3% of childhood NS patients (Christiansen et al., 2014; Kerlin, Ayoob, & Smoyer, 2012b; Kerlin, Smoyer, Tsai, & Boulet, 2015a; Thrombosis: a major contributor to the global disease burden, 2014). Epidemiologic studies have demonstrated that both proteinuria severity and hypoalbuminemia are independently predictive of NS‐related VTE‐risk (Kerlin et al., 2009, 2012b; Lionaki et al., 2012; Mahmoodi, ten Kate, & Waanders, 2008). However, the indications for anticoagulant prophylaxis in the setting of NS remain ill‐defined and controversial (Derebail, Rheault, & Kerlin, 2019; Glassock, 2007; KDIGO Clinical Practice Guideline for Glomerulonephritis, 2012; Kelddal, Nykjaer, Gregersen, & Birn, 2019; Lee et al., 2014). Moreover there is no consensus on when it is safe to discontinue anticoagulation ( Derebail et al., 2019). Virchow's Triad of thrombogenesis includes (a) plasma hypercoagulopathy (the increased tendency of plasma to form blood clots), (b) changes in blood flow (stasis and turbulence), and (3) endothelial dysfunction ( Wolberg et al., 2015). Of these, plasma hypercoagulopathy is thought to be the predominant NS‐associated VTE risk factor (Kerlin, Ayoob, & Smoyer, 2012a; Loscalzo, 2013; Schlegel, 1997; Singhal & Brimble, 2006). We have previously demonstrated, in animal models of NS, that disease severity (proteinuria and hypoalbuminemia) is tightly correlated with endogenous thrombin potential (ETP), a global measure of hypercoagulopathy that is predictive of VTE‐risk, but not yet widely available for clinical use (Ay et al., 2011; Besser, Baglin, Luddington, Vlieg, & Baglin, 2008; Dargaud et al., 2012; Eichinger, Hron, Kollars, & Kyrle, 2008; Emani et al., 2013, 2014; Hron, Kollars, Binder, Eichinger, & Kyrle, 2006; Hylckama et al., 2007, 2015; Kerlin, Waller, et al., 2015; Sonnevi et al., 2013; Tripodi et al., 2007). Therefore, proteinuria and serum albumin levels, which are routinely followed biomarkers of NS disease activity, may become useful surrogate markers of VTE‐risk and thus guide clinical trials of anticoagulant prophylaxis in NS.

Meanwhile, the extent to which disease treatment alters NS‐hypercoagulopathy and, ultimately, modulation of VTE‐risk remains unknown. Current therapeutic options for NS include glucocorticoids (e.g., methylprednisolone; MP) and other immunosuppressive agents, which are associated with significant toxicity and have limited efficacy (Eckardt & Kasiske, 2012; Greenbaum, Benndorf, & Smoyer, 2012; Hodson & Craig, 2008; Longui, 2007; Schonenberger, Ehrich, Haller, & Schiffer, 2011). There is thus a clear need to develop novel NS therapeutics (Greenbaum et al., 2012; Park & Shin, 2011). Glucocorticoids (GC) are thought to modulate their effects primarily via activation of glucocorticoid receptor (GR; *NR3C1)*, which is a member of the nuclear receptor superfamily. We and others have thus investigated thiazolidinediones, which are agonists of an alternative nuclear receptor (peroxisome proliferator‐activated receptor gamma [PPARγ; *PPARG*]) for treatment of NS (Agrawal et al., 2016; Agrawal, Guess, Benndorf, & Smoyer, 2011; Sonneveld et al., 2017). It has previously been demonstrated that pioglitazone (Pio), a thiazolidinedione that is FDA‐approved for treatment of type 2 diabetes mellitus, also reduces proteinuria in rat models of NS (both independently and in combination with MP) (Agrawal et al., 2016; Al‐Majed, Bakheit, Abdel Aziz, Alharbi, & Al‐Jenoobi, 2016; Sonneveld et al., 2017). Some studies suggest that the successful induction of remission may improve or normalize various aspects of the complex hemostatic derangements observed in human NS (Kerlin et al., 2012b). Meanwhile, both MP and Pio may have NS‐independent effects on plasma coagulability (Bodary et al., 2005; Isidori, Minnetti, Sbardella, Graziadio, & Grossman, 2015; Khan et al., 2013; Majoor et al., 2016; Ozsoylu, Strauss, & Diamond, 1962; Pfutzner et al., 2005; Rose, Dunn, Allegret, & Bédard, 2011; Smyth & Jennings, 2005; Zaane et al., 2010). Thus, the ultimate net effects of MP or Pio on NS‐associated hypercoagulopathy remain poorly defined.

Based upon these observations, we hypothesized that efficacious NS therapies that act through nuclear receptor signaling would simultaneously reduce proteinuria, improve hypoalbuminemia, and alleviate hypercoagulopathy. If so, these data may begin to inform the relationship between NS treatment response and VTE‐risk which, in turn, may guide more appropriate and judicious use of anticoagulant prophylaxis in patients with NS. To test this hypothesis, we designed experiments to determine the effects of Pio and MP on ETP, both in rats with puromycin aminonucleoside (PAN) induced nephrosis and in healthy rats (to determine the NS‐independent effects of these drugs on plasma coagulability). We further explored this hypothesis by determining the effects of GC on ETP before and after treatment in a cohort of children with newly‐diagnosed NS. Here we show that nuclear receptor agonist therapies that effectively reduce NS‐associated proteinuria and hypoalbuminemia simultaneously reduce hypercoagulopathy in both PAN‐induced rat NS and childhood NS.

## 2 METHODS

### 2.1 Puromycin Aminonucleoside Rat Nephrosis

The data presented in this report, were derived from both banked samples from our previously reported experiments and additional healthy and PAN‐NS rats (Agrawal et al., 2016). In our previous report, plasma samples for coagulation assays were not collected from all animals. Thus, the data presented herein represent analyses of the animals with available plasma, supplemented with additional animals as required to adequately power the coagulation studies. All procedures were approved by the Institutional Animal Care and Use Committee, in accordance with the NIH Guide for the Care and Use of Laboratory Animals. A summary of the in vivo experiments and their adherence to the ARRIVE Guidelines is provided in Table 1. Male Wistar rats (body weight ~150 g, age ~ 45–50 days) received a single tail vein injection of PAN (50 mg/kg; 5 groups) or saline (4groups) on “Day 0.” PAN‐induced proteinuria was treated daily with sham, Low‐ or High‐dose MP (methylprednisolone; 5 or 15 mg/kg via intraperitoneal injection; hereafter, “Low‐MP” or “High‐MP”), Pio (10 mg/kg via oral gavage), or combination therapy with Pio and Low‐MP (hereafter, “Pio + Low‐MP”), as previously reported, followed by euthanasia 24–28 hr after the final dose (*n* = 8‐13/group) (Agrawal et al., 2016). In order to further confirm the sensitivity of PAN‐NS hypercoagulopathy to High‐MP, we also investigated a range of proteinuria levels obtained by varying the PAN dose and administration route (Kerlin, Waller, et al., 2015; Pippin et al., 2009). For these experiments we used six groups (*n* = 4‐6/ group) of male Wistar rats (weight~150–200 g) which received a single dose of saline (*n* = 8 controls) or PAN, 75 mg/kg via either tail vein (IV) or intraperitoneal (IP) injection, or 100 mg/kg IP. We also compared the effects of High‐MP, Pio, and Pio + Low‐MP treatment in a set of healthy rats not given PAN (*n* = 4/group). Morning spot urine samples were collected on Days 0 (before PAN injection) and 11 for urinary protein:creatinine ratio (UPC) analysis. On day 11, the rats were anesthetized with 3% isoflurane and blood was collected from the inferior vena cava through a 23‐G needle into a final concentration of 0.32% NaCitrate/1.45 µM Corn Trypsin Inhibitor (CTI; Haematologic Technologies Inc., Vermont, VT, USA), processed to Platelet Poor Plasma (PPP) as previously described, and stored at −80ºC until analyzed (Kerlin, Waller, et al., 2015). UPC was measured by Antech Diagnostics (Morrisville, NC), using standard techniques that are fully compliant with Good Laboratory Practice regulations (Kerlin, Waller, et al., 2015). Plasma albumin concentrations were determined using a bromocresol purple (BCP) assay (QuantiChrom BCP; BioAssay Systems, Hayward, CA).

**Table 1:** ARRIVE guidelines for rat experiments

	Total Rats	*N* per Group	PAN Dose	Treatments	ARRIVE Guidelines
Main Study Rats	57	8–13	50 mg/kg, IV	Pio, Low‐ & High‐MP, Pio + Low‐MP	Randomized groups; Investigators performing sample collection and assays blinded
Supplemental Rats	38	4–8	75 mg/kg, IP&IV 100 mg/kg, IP	High‐MP	
Non‐Nephrotic Rats	12	4	None	High‐MP, Pio, Pio + Low‐MP	

Note

IP, intraperitoneal injection; IV, intravenous tail vein injection; Low‐ and High‐MP, 5 and 15 mg/kg methylprednisolone, respectively; Pio, 10 mg/kg pioglitazone via oral gavage; ARRIVE, Reporting In Vivo Experiments.

### 2.2 Pediatric nephrology research consortium cohort

Children with incident NS were recruited from Pediatric Nephrology Research Consortium (PNRC) participating centers (see participating PNRC centers and investigators in Appendix). The study protocols and consent documents were approved by the Nationwide Children's Hospital Institutional Review Board (IRB05‐00544, IRB07‐004, & IRB12‐00039) and at each participating PNRC center. Glucocorticoid (GC) therapy naïve children 1–18 years of age presenting with edema and proteinuria ≥ 3+ by dipstick were eligible for enrolment. Steroid‐sensitive NS (SSNS) was defined as disease remission following 7 (± 0.4) weeks of standard‐of‐care GC therapy (dose and formulation at the treating physician's discretion), whereas steroid‐resistant NS (SRNS) was defined as failure to achieve complete remission during this time frame, as determined by resolution of proteinuria by urine dipstick or UPC. Blood was collected at the time of enrolment (prior to GC exposure) and a paired blood specimen was obtained after 7 (± 0.4) weeks of GC therapy at the time steroid‐responsiveness was assessed. Importantly, all of the children were still on GC therapy when the second sample was obtained. Blood was collected into 0.1 M sodium citrate cell preparation tubes containing a cell separator system (BD Vacutainer CPT (REF 362,761) Becton, Dickinson and Company, Franklin Lakes, NJ) at an 8:1 blood‐to‐citrate ratio (~0.33% final concentration NaCitrate). After processing, plasma was frozen at −80°C and transferred to the Abigail Wexner Research Institute at Nationwide Children's for analysis. Select demographics from this pediatric cohort at disease presentation are provided in Table 2.

**Table 2:** Childhood nephrotic syndrome cohort demographics

	SSNS (*n* = 24)	SRNS (*n* = 14)
Age^a^ (y); mean ± SE^b^	5.74 ± 0.78	9.55 ± 1.03
Sex^a^; *n* (%)		
Male	12 (50)	5 (35.7)
Female	11 (45.8)	9 (64.2)
Race; *n* (%)		
White	10 (41.7)	6 (42.9)
Black/African American	5 (20.8)	6 (42.9)
Other	6 (25)	1 (7.1)
Not Reported	3 (12.5)	1 (7.1)
BMI^a^; Percentile ± SE	86.7 ± 3.45	89.1 ± 3.11
Weeks of GC Therapy; mean ± SE	7.0 ± 0.6	7.0 ± 0.6

Abbreviations: BMI, body mass index; GC, glucocorticoid; SSNS, Steroid‐Sensitive Nephrotic Syndrome; SRNS, Steroid‐Resistant Nephrotic Syndrome

^a^ Age, sex, and BMI not reported for 1 patient;

^b^ *p* < .05.

### 2.3 Coagulation parameters

ETP was determined on PPP (neat and diluted 1:1 with buffer, for patients and rats, respectively) using the Technothrombin TGA kit (Technoclone, Vienna, Austria) and TGA RC low reagent, and read on a Spectramax M2 fluorescent plate reader (Molecular Devices, Sunnyvale, California), as previously described (Kerlin, Waller, et al., 2015). To confirm sample stability and assay validity, TGA was performed on previously unthawed PPP aliquots and both biobanked and new samples were run simultaneously. Plasma antithrombin (AT) concentrations were measured by ELISA (rat Antithrombin III ELISA kit, MyBiosource, San Diego, CA). Plasma prothrombin concentration was measured by gel electrophoresis and immunoblotting, as follows: Equal amounts of PPP were diluted in Laemmli buffer (BIO‐RAD, Hercules, CA) with β‐mercaptoethanol, resolved on a 10% SDS‐polyacrylamide gel (Mini‐PROTEAN II, BIO‐RAD) and then electrophoretically transferred to a polyvinylidene fluoride membrane (Millipore, Billerica, MA). After blocking with 5% nonfat dry milk solution, membranes were incubated overnight at 1:2000 in primary antibody (Anti‐murine Prothrombin, Haematologic Technologies Inc, Essex Junction, VT) followed by corresponding secondary antibody conjugated to horseradish peroxidase. Quantitative determination of protein was performed by autoradiography after revealing the antibody‐bound protein by enhanced chemiluminescence reaction (MilliporeSigma, Burlington, MA). The density of the bands on scanned autoradiographs was quantified relative to an identical volume of rat pooled normal plasma using ImageJ (NIH, Bethesda, MD). ELISA and immunoblot antibodies were validated using species‐specific positive (purified species‐specific protein; Haematologic Technologies, Inc, Essex Juntion, VT) and nonspecific protein negative controls. Plasma AT activity was measured using a modified amidolytic method as described previously (Kerlin, Waller, et al., 2015; Ødegård, 1975), while plasma prothrombin functional activity was determined using a commercially available chromogenic assay (Rox Prothrombin; DiaPharma, West Chester Township, OH).

### 2.4 Statistical analyses

The unpaired Student's *t*‐test was used for single comparisons and one‐ or two‐way ANOVA (analysis of variance) for multiple group comparisons, using SigmaStat software (Systat, San Jose, CA). When a significant difference was identified by ANOVA, post hoc tests were performed using the Student–Newman–Keuls technique. Chi‐square or Fisher's exact test, as appropriate, were used for categorical comparisons. Statistical significance was defined as *p < *.05. Data are presented as mean ± SE.

## 3 RESULTS

### 3.1 Both methylprednisolone and pioglitazone alleviate proteinuria and hypoalbuminemia

**Figure 1:**
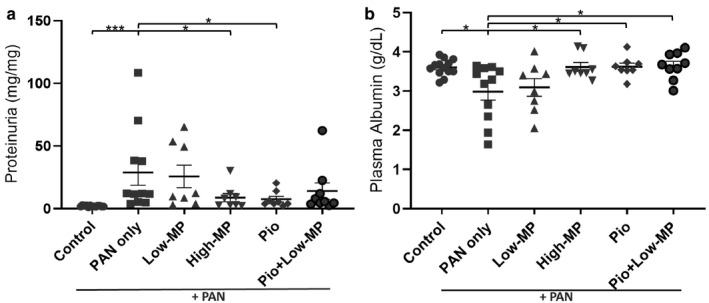
Both Methylprednisolone and Pioglitazone Alleviate Proteinuria and Hypoalbuminemia. Mean ± SE of (a) proteinuria and (b) plasma albumin concentration in a PAN‐induced rodent model of NS, with/without concomitant methylprednisolone (MP) and/or pioglitazone (Pio) treatment (*n* = 8‐13/group). *p < .05 ***p < .001

As expected, significant levels of proteinuria and hypoalbuminemia were observed 11 days post‐PAN (Figure 1). Treatment with High‐MP, Pio, or Pio + Low‐MP partially ameliorated PAN‐NS. The High‐MP and Pio groups had significantly reduced proteinuria compared to untreated PAN rats (*p < *.05), whereas the Low‐MP and Pio + Low‐MP groups did not improve. Intriguingly, Pio and High‐MP similarly improved proteinuria (73.9% and 69.6% reductions versus. sham, respectively). As expected, plasma albumin levels improved in concert with proteinuria improvement. While Low‐MP did not improve hypoalbuminemia, High‐MP, Pio, and Pio + Low‐MP all improved albumin levels versus no treatment (*p < *.05).

### 3.2 Hypercoagulopathy improves in parallel with nephrosis following treatment

**Figure 2:**
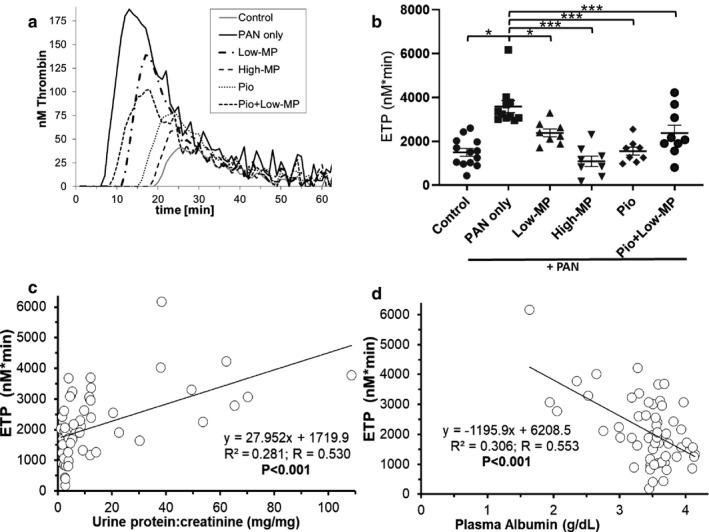
Hypercoagulopathy Improves in Parallel with Nephrosis Following Treatment. (a) Representative thrombin generation graphs from one individual rat per group, and graph of mean ± SE for (b) endogenous thrombin potential (ETP), in a PAN‐induced rodent model of NS, with/without concomitant methylprednisolone (MP) and/or pioglitazone (Pio) treatment (*n* = 8‐13/group). (c‐d) Linear regression analysis correlating disease severity (proteinuria (c) and hypoalbuminemia (d)) with ETP. *p < .05 ***p < .001

In addition to ETP, several other parameters are derived from thrombin generation assays; however, ETP was consistently the most strongly correlated with NS severity (Table 3). As previously demonstrated, proteinuria (*p* < .001) and hypoalbuminemia (*p < *.001) were significantly correlated with ETP (Figure 2) (Kerlin, Waller, et al., 2015). Successful treatment with either High‐MP or Pio reduced ETP to levels similar to control (*p* < .001 versus. PAN). In contrast, Low‐MP and Pio + Low‐MP significantly reduced ETP versus. PAN (*p < *.05 and *p* < .001, respectively), but they did not correct ETP to control values, representing a partial ETP recovery.

**Table 3:** Correlation of associated thrombin generation assay parameters with nephrotic disease severity

TGA Parameter	Main Study Rats		Supplemental Rats		Nephrotic Syndrome Patients	
	Proteinuria	Albuminemia	Proteinuria	Albuminemia	Proteinuria	Albuminemia
ETP (nM*min)	*R* = 0.530; *p* < .001	*R*=‾0.553; *p* < .001	*R* = 0.830; *p* < .001	*R*=‾0.793; *p* < .001	*R* = 0.254; *p* = .040	*R*=‾0.562; *p* < .001
Peak Thrombin (nM)	*R* = 0.279; *p* = .035	*R*=‾0.315; *p* = .017	*R* = 0.642; *p* < .001	*R*=‾0.600; *p* < .001	*R* = 0.349; *p* = .011	*R*=‾0.423; *p* < .001
Velocity Index (nM/min)	*R* = 0.032; *p* = .788	*R*=‾0.071; *p* = .611	*R* = 0.391; *p* = .020	*R*=‾0.264; *p* = .126	*R* = 0.367; *p* = .007	*R*=‾0.315; *p* = .006
Lag Phase (min)	*R* = 0.055; *p* = .674	*R*=‾0.063; *p* = .629	*R* = 0.551; *p* < .001	*R*=‾0.532; *p* = .001	*R* = 0.167; *p* = .236	*R*=‾0.032; *p* = .836

Abbreviations: ETP, Endogenous Thrombin Potential; TGA, Thrombin Generation Assay.

### 3.3 Qualitative antithrombin deficit and treatment response

**Figure 3:**
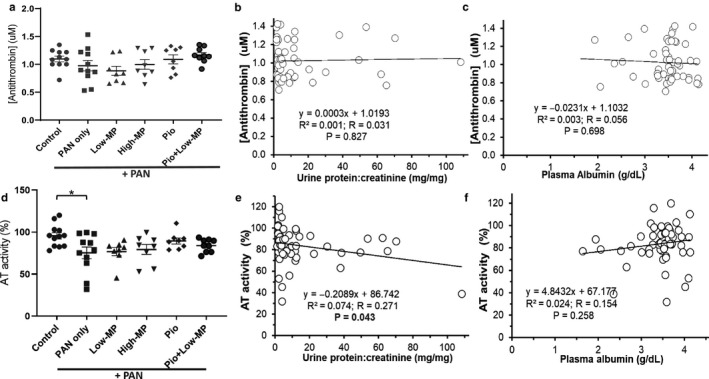
Qualitative Antithrombin Deficit and Treatment Response. Mean ± SE of plasma AT concentration (a‐c) and AT activity (d‐f) in PAN‐NS, with/without methylprednisolone (MP) and/or pioglitazone (Pio) treatment, and linear regression analysis with proteinuria (b, e) and hypoalbuminemia (c, f) (*n* = 8‐13/group). There was no correlation between ETP and AT activity or AT concentration (*p* = .066 and *p* = .186, respectively; data not shown). *p < .05

**Figure 4:**
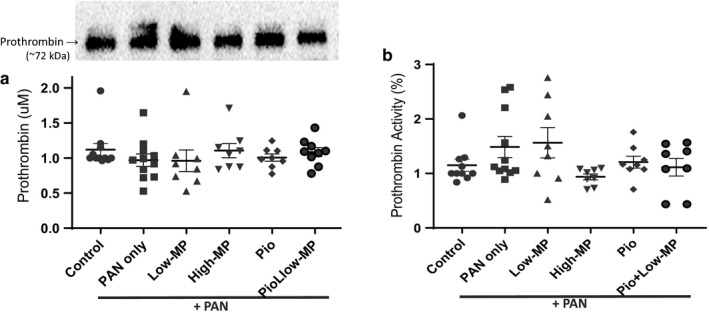
Prothrombin Antigen and Activity are not Altered by PAN‐Induced Nephrosis or Treatment. Mean ± SE of plasma prothrombin concentration (a) and activity (b) in PAN‐induced rodent model of NS, with/without concomitant methylprednisolone (MP) and/or pioglitazone (Pio) treatment (*n* = 8‐13/group)

We previously demonstrated a qualitative antithrombin (AT) deficiency in PAN‐NS (Kerlin, Waller, et al., 2015). As expected, based on this prior observation, plasma AT antigen (protein) levels were unaffected by either disease or treatment whereas AT inhibitory activity was significantly correlated with UPC (*p* = .043) but not plasma albumin (*p* = .258; Figure 3). Because ETP was significantly corrected with treatment and acquired AT deficiency is thought to be a major mechanism underlying NS‐hypercoagulopathy (Kerlin et al., 2012b), we were surprised to find that none of the treatments significantly improved AT activity. Nonetheless, AT activity was no longer significantly lower than control values, suggesting a modest degree of partial correction. Moreover there was no significant correlation between ETP and AT activity or antigen (*p* = .066 and *p* = .186, respectively; data not shown). There were also no significant differences in prothrombin antigen or activity levels by treatment group (Figure 4).

### 3.4 Disease and hypercoagulopathy responses persist across a broad range of disease severity

**Figure 5:**
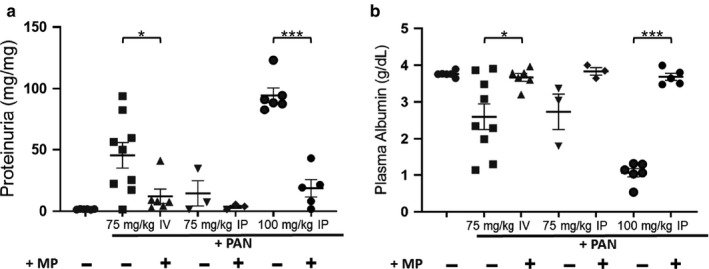
PAN Nephrotic Rats Are Responsive to High‐Dose Methylprednisolone Treatment. Mean ± SE of (a) proteinuria and (b) plasma albumin concentration in a PAN‐induced nephrotic rats (*n* = 4‐8/group). Varying disease severity was induced in male Wistar rats by a single intravenous (IV) or intraperitoneal (IP) injection of 75 or 100 mg/kg PAN. Steroid sensitivity was confirmed after 11 days of treatment with either high‐dose methylprednisolone (+MP; 15 mg/kg) or sham saline (‐). *p < .05 ***p < .001

**Figure 6:**
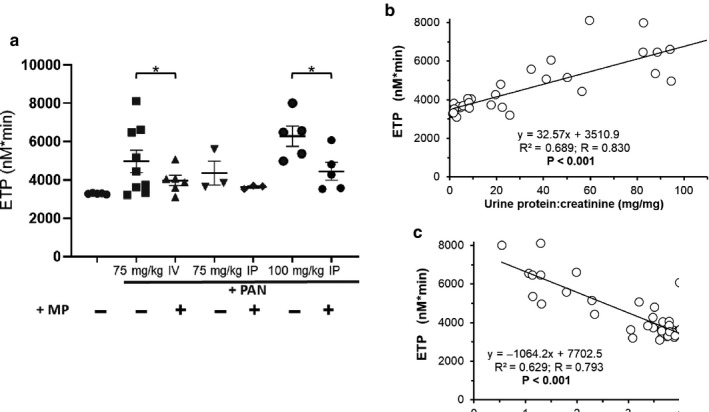
High‐Dose Methylprednisolone Corrects Thrombin Generation in More Severe PAN‐Induced Nephrosis. Mean ± SE of Endogenous Thrombin Potential (a) in rats made nephrotic with a single intravenous (IV) or intraperitoneal (IP) injection of 75 or 100 mg/kg PAN injection, and then treated with high‐dose methylprednisolone (+MP; 15 mg/kg) or sham saline (‐) for 11 days (*n* = 4‐8/group). ETP was significantly correlated with proteinuria and hypoalbuminemia (b, c). *p < .05

**Figure 7:**
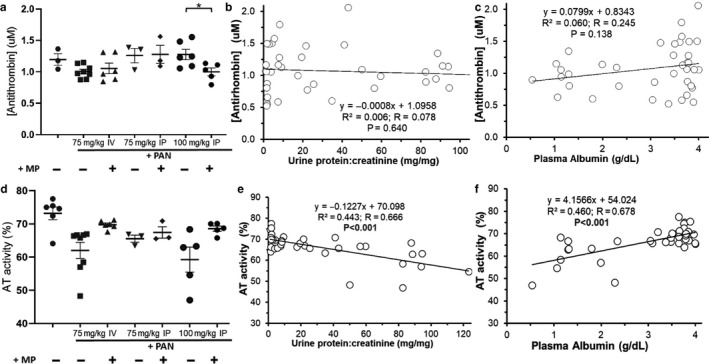
Qualitative Antithrombin Deficit Persists in More Severe PAN‐Induced Nephrosis. Mean ± SE of plasma antithrombin (AT) antigen (a‐c) & AT activity (d‐f) in a PAN‐induced rodent model of NS treated with high‐dose methylprednisolone (+MP; 15 mg/kg) or sham saline (‐) for 11 days (*n* = 4‐8/group). Plasma AT antigen and activity were significantly correlated with disease severity. *p < .05 **p < .01

Rat PAN‐NS severity is variable in a manner dependent on PAN dose and route of administration (Kerlin, Waller, et al., 2015; Pippin et al., 2009). We thus assessed the hypercoagulopathy response across a range of PAN‐NS severities. As expected, low‐dose IP PAN (75 mg/kg) produced only marginal, insignificant levels of proteinuria and hypoalbuminemia (Pippin et al., 2009). However, plasma albumin and UPC were responsive to High‐MP treatment following higher doses of PAN (75 mg/kg IV or 100 mg/kg IP) (Figure 5). Similarly, High‐MP treatment improved ETP toward control values under these more severely nephrotic conditions (Figure 6). ETP was again significantly correlated with PAN‐NS severity. As expected, there were no significant differences in AT antigen levels across the PAN‐NS severity spectrum, nor were antigen levels correlated to proteinuria or plasma albumin (Figure 7). While AT activity was significantly correlated with markers of NS disease severity, it was not consistently improved by High‐MP. Again, there was no correlation between ETP and AT activity (*p* = .435; data not shown).

**Figure 8:**
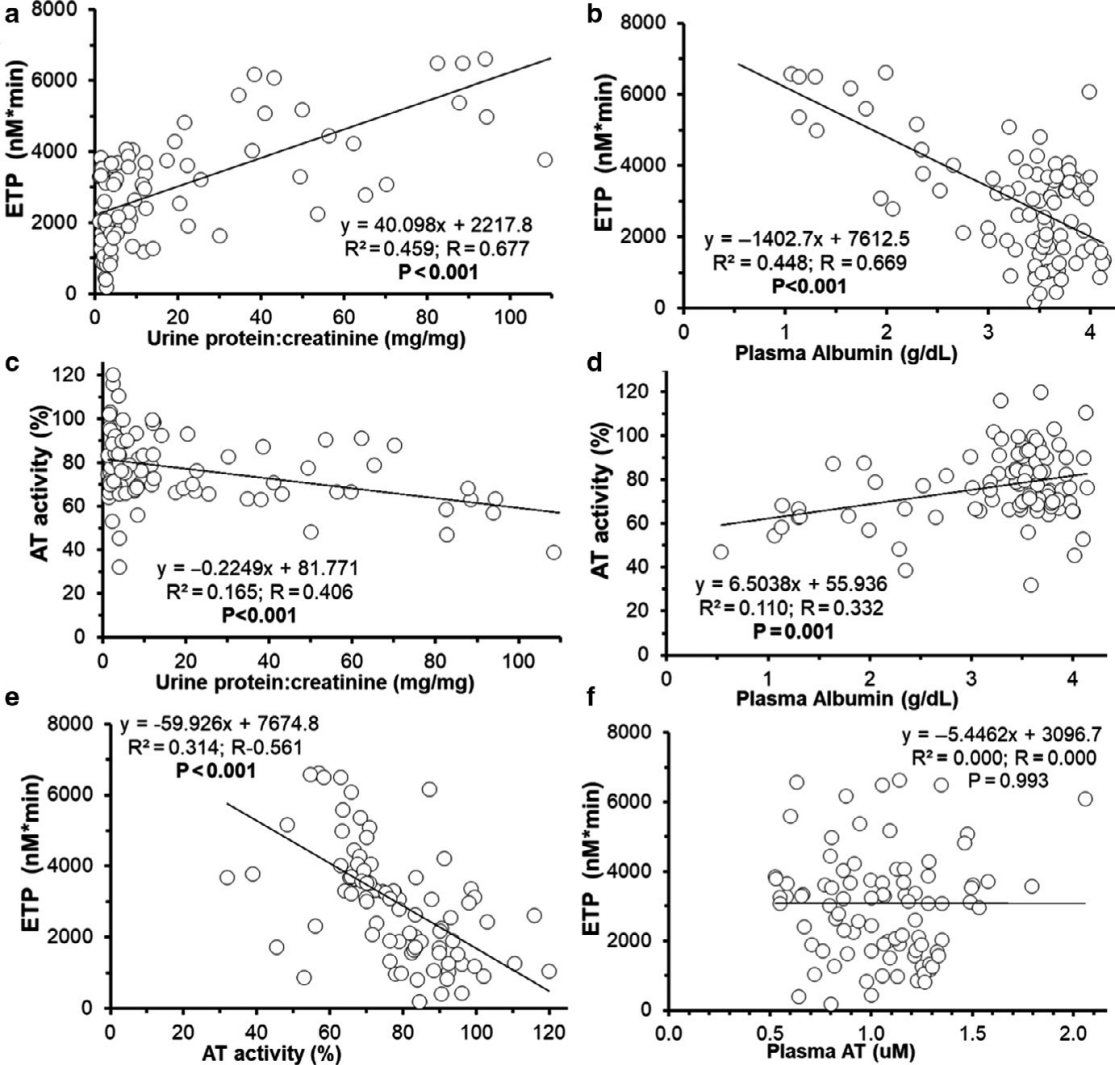
Hypercoagulopathy Responses Persist Across a Broad Range of Disease Severity. Linear regression analysis of disease severity (proteinuria (a, c) and hypoalbuminemia (b, d)) and coagulation markers (ETP (a, b), AT activity (c, d)), in all PAN‐NS rats combined (*n* = 95). (e, f) Linear regression analysis of the relationship between ETP and AT activity (e) and AT concentration (f) in the combined rat groups (*n* = 95)

To further evaluate the hypothesis that NS hypercoagulopathy correlates directly with NS‐severity, we performed linear regression analyses on the combined data from all PAN‐NS experimental groups presented in this paper (Figure 8). As expected, in this large combined dataset, proteinuria and hypoalbuminemia were correlated with ETP (*R* ^2^ = 0.459 and 0.448, respectively; *p* < .001). Plasma AT activity was also significantly correlated to proteinuria and hypoalbuminemia severity (*R* ^2^ = 0.165, *p* < .001 and *R* ^2^ = 0.110, *p* = .001; respectively). In contrast to the smaller analyses of each PAN‐NS cohort where there was no relationship between AT activity and ETP, in the combined analysis a significant correlation between AT activity and ETP emerged (*R* ^2^ = 0.314, *p* < .001; Figure 8e). However, there was still no significant relationship between ETP and AT antigen concentration (*p* = .993).

### 3.5 Both methylprednisolone and pioglitazone induce hypercoagulopathy in non‐nephrotic rats

**Figure 9:**
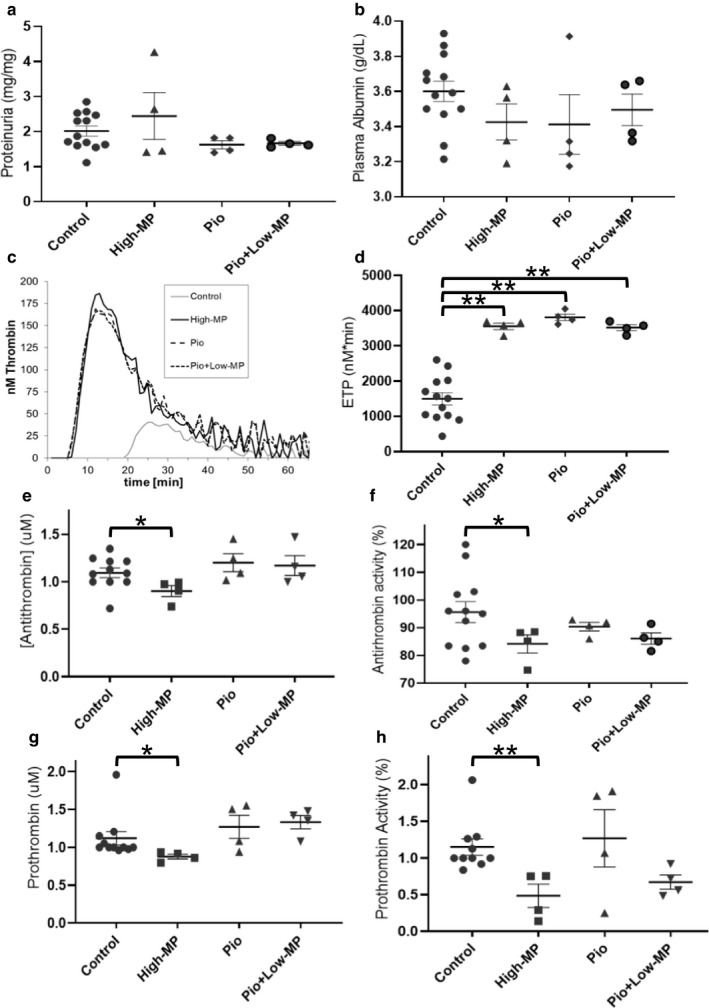
Healthy Rats Treated with Methylprednisolone and Pioglitazone Alone or in Combination Exhibit Elevated Endogenous Thrombin Potential and Altered Coagulation Parameters. Mean ± SE of (a) proteinuria, (b) plasma albumin concentration, (c) Representative thrombin generation graphs from one individual rat per group, (d) mean ± SE Endogenous Thrombin Potential, (e) antithrombin antigen (f) and activity, (g) prothrombin antigen (h) and activity, in healthy Wistar rats treated with saline (Control), high‐dose methylprednisolone (MP; 15 mg/kg), pioglitazone (Pio), or combination therapy (Pio + Low‐GC) (*n* = 4‐13/group). *p < .05 **p < .01

As expected, there was no change in proteinuria or plasma albumin, in healthy rats treated with High‐MP, Pio, or Pio + Low‐MP (Figure 9). However, all three of these treatments significantly increased ETP in healthy rats (*p* < .01; Figure 9d). As expected, in these otherwise healthy animals without PAN‐NS, ETP was not correlated with proteinuria or plasma albumin levels (data not shown). Interestingly, however, healthy animals given High‐MP also exhibited decreased AT antigen, AT activity, prothrombin antigen, and prothrombin activity (*p < *.05; Figure 9e‐H). In contrast, Pio and Pio + Low‐MP did not significantly change the antigen or activity values for either AT or prothrombin. AT activity was not correlated to AT protein levels (*p* = .841) or ETP (*p* = .118) in these healthy rats (data not shown).

### 3.6 Hypercoagulopathy improves in children with steroid‐sensitive, but not steroid‐resistant nephrotic syndrome

**Figure 10:**
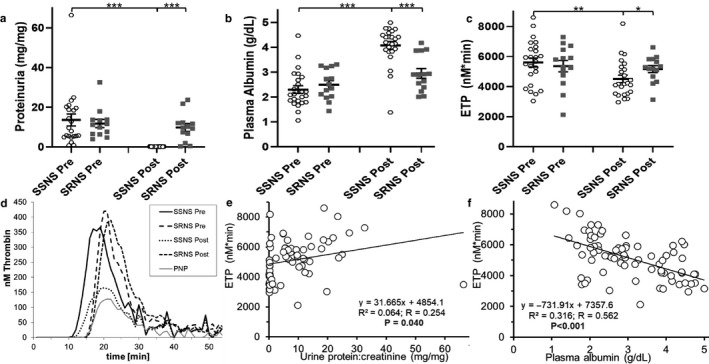
Hypercoagulopathy Improves in Children with Steroid‐Sensitive, but not Steroid‐Resistant Nephrotic Syndrome. Mean ± SE of proteinuria (a), plasma albumin (b), and ETP (c) in childhood steroid‐sensitive NS (SSNS; *n* = 24) and steroid‐resistant NS (SRNS; *n* = 14) at disease presentation (“Pre”) and following glucocorticoid treatment (“Post”). (d) Representative thrombin generation graphs from one individual per group and a human pooled normal plasma (PNP) control. (e, f) Linear regression analysis correlating ETP with proteinuria and hypoalbuminemia, respectively. *p < .05 **p < .01 ***p < .001

Thirty‐eight children were enrolled in the PNRC cohort (24 with SSNS and 14 with SRNS; Table 2). As expected (Eddy & Symons, 2003), the children with SRNS were significantly older at presentation, otherwise the groups were demographically similar. Genetics are known to play a role in steroid‐responsiveness (Adeyemo et al., 2018; Asharam et al., 2018; Govender et al., 2019; Gribouval et al., 2018, 2019). However, while genetic and other mechanisms underlying steroid‐responsiveness in this small cohort were not evaluated in the present study, they have been the subject of previously reported biomarker studies (Agrawal et al., 2020; Gooding et al., 2020). Importantly, none of the children in this small cohort were diagnosed with venous thromboembolism, which occurs in ~3% of childhood NS cases (Kerlin et al., 2012a). Proteinuria, plasma albumin, and ETP were not different between the SSNS and SRNS groups at disease presentation (before GC treatment; Figure 10). By definition, GC therapy significantly improved proteinuria and hypoalbuminemia in children with SSNS (*p* < .001). ETP was significantly reduced at the follow‐up visit for children with SSNS in comparison to their ETP at presentation (4,505 ± 251 versus. 5,608 ± 298 nM*min, *p* < .01). In contrast, there was no significant change in ETP for children with SRNS (5,362 ± 377 versus. 5,165 ± 233 nM*min at presentation and follow‐up, respectively; *p* = .661). When combining the disease activity for all these children from both visits, ETP was significantly correlated with both proteinuria (*p* = .04) and plasma albumin (*p* < .001) suggesting that hypercoagulopathy is correlated with disease activity in children with NS.

## 4 DISCUSSION

This study investigated the effects of two nuclear receptor agonists, which have disparate mechanisms of action, on endogenous thrombin potential (ETP), a biomarker of venous thromboembolism (VTE) risk, in a well‐established rodent nephrotic syndrome (NS) model as well as in children with idiopathic NS. Both methylprednisolone (MP), an established frontline childhood NS therapy, and pioglitazone (Pio; an investigational NS therapeutic) significantly reduced proteinuria in rats with puromycin aminonucleoside (PAN) nephrosis and simultaneously improved NS‐associated hypercoagulopathy, as measured by ETP. Importantly, Pio was as effective as High‐dose MP, suggesting that it may enable a steroid‐sparing treatment strategy. These studies further demonstrate that NS disease activity (as determined by proteinuria and hypoalbuminemia) is correlated with ETP, independent of treatment modality. Moreover, in this cohort of children with NS, there was a significant ETP reduction in those with steroid‐sensitive NS (SSNS), whereas those with steroid‐resistant NS (SRNS) had no ETP improvement from their baseline values. Collectively, these data suggest that treatments which effectively reduce proteinuria may simultaneously reduce NS‐associated VTE‐risk. Moreover because ETP is correlated with disease activity, even a partial reduction in proteinuria may provide a secondary clinical benefit by reducing NS‐associated hypercoagulopathy‐mediated VTE‐risk. Finally, this study provides evidence that consideration should be given for inclusion of ETP (and/or other VTE‐risk biomarkers) as a component of composite outcomes when evaluating investigational NS therapeutics in future clinical trials.

These experiments provide novel evidence that NS‐associated hypercoagulopathy improves in parallel to NS‐treatment response and simultaneously confirm our previous observation that the hypercoagulopathy is proportional to disease activity (Kerlin, Waller, et al., 2015). The improvements in ETP were seen using two drugs which act through different members of the nuclear receptor superfamily (MP via GR and Pio via PPARγ) (Agrawal et al., 2011, 2016). Interestingly, both of these agents enhanced ETP in non‐nephrotic rats. Thus, these data strongly suggest that the benefits of these drugs to diminish NS‐associated hypercoagulopathy are an indirect consequence of proteinuria (glomerular filtration defect) reduction. We have previously reported on the molecular mechanisms underlying the antiproteinuric effects of both GC and Pio (Agrawal et al., 2011, 2016; Ransom, Lam, Hallett, Atkinson, & Smoyer, 2005a; Ransom, Vega‐Warner, Smoyer, & Klein, 2005b). Nonetheless, because nuclear receptors act as transcription factors to alter expression of broad gene sets, additional studies should focus on how these therapies alter both glomerular filtration‐ and hemostasis‐relevant gene expression. For example, these data suggest that MP, but not Pio, may decrease AT (*Serpinc1*) expression in healthy rats. Furthermore, these data suggest the possibility that any treatment that effectively reduces NS severity may also indirectly ameliorate NS‐associated hypercoagulopathy. This concept is further supported by the human data showing that children with SSNS had improved ETP from their baseline values, whereas children with SRNS did not. Thus, an indirect benefit of effective NS therapy is expected to be reversal of hypercoagulopathy and thus, decreased VTE‐risk. These data, using a global hemostasis assay are consistent with previous studies demonstrating improvement of individual coagulation system protein and activity levels between active NS and NS in remission (Al‐Mugeiren, Abdel Gader, Al‐Rasheed, & Al‐Salloum, 2006; Brummel‐Ziedins & Wolberg, 2014; Citak, Emre, Sirin, Bilge, & Nayir, 2000; Ueda, 1990; Ueda et al., 1987).

NS complications may occur due to the disease itself or consequent to therapeutic side effects. Glucocorticoids (i.e., MP and prednisolone) remain the most common frontline therapy for idiopathic childhood NS, but are associated with well‐recognized and potentially serious side effects (Greenbaum et al., 2012; Park & Shin, 2011). In addition, ~10%–15% of childhood NS cases are steroid‐resistant (SRNS) and some data suggest that the prevalence of SRNS is rising ( Banaszak & Banaszak, 2012). Moreover NS continues to be a leading cause of ESKD despite GC and alternative immunosuppressant therapies (Eckardt & Kasiske, 2012; Greenbaum et al., 2012; Hodson & Craig, 2008; Schonenberger et al., 2011; Yaseen et al., 2017). Thus, a critical need remains to develop more targeted and less toxic therapies to improve NS outcomes. In this regard, we previously demonstrated that Pio reduced proteinuria in PAN‐induced NS (both independently and in combination with reduced‐dose MP), suggesting that Pio may enable a steroid‐sparing NS treatment strategy (Agrawal et al., 2016). The present study confirms and extends these observations to additional PAN‐NS groups and demonstrates that Pio treatment (alone or in combination with low‐dose MP) results in a partial proteinuria reduction of (73.9% or 51.4%, respectively) that was similar to conventional, high‐dose MP (69.6%). In contrast, low‐dose MP without Pio was not beneficial. Furthermore, this study is the first to describe the effects of Pio treatment on hemostasis in NS, demonstrating that effective proteinuria reduction correlated with a complete alleviation of NS‐associated hypercoagulopathy, as evidenced by ETP correction to values indistinguishable from those of control rats. Thus, Pio (alone or in combination) successfully reduced both proteinuria and NS‐associated hypercoagulopathy. Therefore, Pio may have significant potential as a novel NS therapy, to simultaneously reduce disease severity (primary outcome) and limit both steroid‐related side effects and VTE‐risk (secondary outcomes). Pio may be particularly beneficial for childhood NS, where there is less risk of thiazolidinedione‐mediated heart failure (Cheng, Gao, & Li, 2018; Jearath, Vashisht, Rustagi, Raina, & Sharma, 2016; Trachtman et al., 2015). Thus, a multicenter randomized controlled trial assessing the clinical benefits of Pio in the treatment of childhood idiopathic NS should include ETP as a component of the composite outcome.

It has long been suggested that GC administration induces procoagulant effects in otherwise healthy individuals (Ozsoylu et al., 1962). Although studies in healthy volunteers are appropriate for detecting unconfounded effects of GC, in clinical practice GC are most commonly used for inflammatory diseases and in surgical settings, and thus most published literature on the effects of MP on hemostasis are confounded by the simultaneous effects of the underlying disease processes (Isidori et al., 2015; Johannesdottir et al., 2013; Lilova, Velkovski, & Topalov, 2000; Zaane et al., 2010). Nonetheless, there appears to be little doubt that patients treated with GC have higher VTE‐risk. For example, a recent, large population‐based case–control study found that systemic GC therapy (including MP) was associated with increased VTE‐risk, an observation that persisted after adjustment for underlying disease severity, for both inflammatory and non‐inflammatory conditions (Johannesdottir et al., 2013). Results from previous studies suggest that alterations in coagulation balance are dependent on both GC drug and dose. A variety of hemostatic effects have been detected including: increased platelet aggregation, shortened partial thromboplastin times, increased levels of factors V, VII, VIII, XI, and fibrinogen, which are associated with enhanced arterial thrombosis in vivo (Isidori et al., 2015; Majoor et al., 2016; Ozsoylu et al., 1962; Rose et al., 2011; Zaane et al., 2010). However, data regarding the effects of GC on overall hemostatic balance, as determined by ETP or other global coagulation assays are lacking. Only one previous study employed a thrombin generation assay in healthy adults administered prednisolone (0.5 mg kg^‐1^ day^‐1^ orally) for 10 days (Majoor et al., 2016). That investigation revealed increases in peak thrombin and velocity‐index, but not ETP. However, there were unexpectedly high thrombin generation parameters in the placebo group (at baseline) that limited the interpretation of the data. In this study, healthy rats treated with MP were hypercoagulable by ETP. In addition, both qualitative and quantitative AT defects were present in healthy rats treated with High‐MP, but not with Pio or Pio + Low‐MP therapy. Taken together the present and previously published data suggest that GCs induce a procoagulant state in healthy subjects.

In contrast to the procoagulant effects of GC, thiazolidinediones may have anticoagulant effects that include decreased factor VII, plasminogen activator inhibitor‐1, von Willebrand factor, and platelet activation (Bodary et al., 2005; Khan et al., 2013; Pfutzner et al., 2005; Smyth & Jennings, 2005). In healthy rats administered low (1 mg/kg) or high (10 mg/kg) dose Pio for 10 days, decreased platelet aggregation and delayed arterial thrombus formation were observed (Li et al., 2005). These beneficial effects were likely secondary to increases in aortic wall expression of thrombomodulin and constitutively‐expressed nitric oxide synthase (cNOS). In contrast to these anticoagulant effects, the present data demonstrate that both Pio and Pio + Low‐MP increase thrombin generation (ETP) when administered to healthy animals. However, in vivo thrombosis modeling suggests that PPARγ signaling is antithrombotic, implying that the antithrombotic effects of Pio on the vessel wall likely dominate the prothrombotic ETP elevations found in the plasma compartment (Jin et al., 2015; Pelham, Keen, Lentz, & Sigmund, 2013). Consistent with this interpretation, the clinical evidence to date suggest that Pio has an overall positive safety profile and lack of VTE‐risk (Erdmann et al., 2007; Wilcox, Kupfer, & Erdmann, 2008).

Although it has been suggested that GC therapy may contribute to increased VTE‐risk during NS, results from epidemiologic studies have generally failed to support a clear link (Kerlin et al., 2009; Lilova et al., 2000; Mehls, Andrassy, Koderisch, Herzog, & Ritz, 1987; Singhal & Brimble, 2006). Whereas SRNS patients are known to have a higher VTE‐risk than those with SSNS (Lilova et al., 2000; Ulinski et al., 2003), the factors contributing to this discrepancy are not yet fully elucidated. However, the present data demonstrating that the acquired NS‐hypercoagulopathy is highly correlated with disease activity, during both disease and treatment, suggest that the persistent proteinuria associated with SRNS corresponds with persistent hypercoagulopathy. Indeed, the ETP data from this small cohort of children with SSNS versus. SRNS supports this hypothesis but should be extended to a larger patient cohort before reaching definitive conclusions. If confirmed, this paradigm would further support the idea that patients with persistent NS should remain on anticoagulant prophylaxis whereas it may be safe to discontinue anticoagulation in patients who have achieved a sustained complete remission (Derebail et al., 2019; Monagle et al., 2018).

We previously demonstrated a qualitative antithrombin (AT) deficiency in PAN‐NS that was confirmed in the present study (Kerlin, Waller, et al., 2015). AT activity did not significantly improve in the time range studied, despite successful therapeutic responses in these animals. However, post‐therapeutic AT activities were no longer significantly different than either control values or untreated PAN‐NS values, suggestive of a partial improvement in response to therapy. Additional studies with later time points may reveal a gradual improvement in AT activity levels that lags behind of proteinuria, albumin, and ETP improvements. This observation also suggests that the acquired AT deficiency of NS is not mechanistically responsible for the observed hypercoagulopathy. Consistent with this postulate, there was no direct correlation between AT activity and ETP in the individual rat experiments (*n* = 58 & 38). Only when combining all rat groups together (*n* = 95) was a correlation between ETP and AT activity uncovered, suggesting that in PAN‐NS, the qualitative AT deficit likely makes only a minor contribution to increased thrombin generation. This interpretation is consistent with the appearance of the thrombin generation curves wherein the ETP increases appear to originate from shortened lag‐times and increased peak thrombin values, rather than the tail prolongation that has been demonstrated in AT deficient plasma (Miyawaki et al., 2012). Plasma AT consists of two glycoforms (fully glycosylated α‐AT (>90%) and hypoglycosylated β‐AT (<10%); in comparison to α‐AT, β‐AT binds heparin more tightly, is cleared more rapidly from circulation, and is the predominant glycoform found in subendothelial spaces (Picard, Ersdal‐Badju, & Bock, 1995). Thus, NS‐mediated dysregulation of AT glycosylation or clearance may explain the observed qualitative AT deficiency. However, we have been unable to demonstrate a shift in AT glycoforms in PAN‐NS or human NS plasma samples (data not shown). Thus, while the mechanism underlying this qualitative deficiency remains mysterious, these data suggest that it is only a minor contributor to hypercoagulopathy as measured by plasma ETP. Whether shifts in the α‐AT to β‐AT ratio in the subendothelial compartment may contribute to NS‐associated VTE remains to be determined.

In these experiments, variations in the single‐dose PAN‐NS model were used to investigate treatment effects at peak disease severity (day 11), therefore Pio and/or MP treatment was commenced on the same day as PAN administration (before the onset of proteinuria), whereas in the clinical setting treatment is begun only after the onset of symptomatic proteinuria. Thus, future studies investigating the efficacy of Pio and/or MP to reduce established proteinuria in PAN‐NS and other NS models are warranted to further elucidate the potential benefits of these treatments on NS‐associated hypercoagulopathy. Nonetheless, the data demonstrating improved ETP with high‐dose MP across a wide range of proteinuria severity suggests that the relationship between disease reduction and ETP improvement will be generalizable to other models. Moreover the proteinuria levels induced in the Pio experiments are on par with those we previously demonstrated to exacerbate thrombosis in PAN‐NS (Kerlin, Waller, et al., 2015), therefore the Pio‐induced reductions in ETP are likely to be physiologically relevant. Hepatic synthesis of coagulation proteins following PAN administration has not yet been directly studied, thus there may be direct effects of PAN‐NS on coagulation status. However, the hypercoagulopathy of PAN‐NS closely resembles that seen in other experimental NS models and human NS patients, suggesting that the hypercoagulopathy is secondary to nephrosis as opposed to off‐target coagulation effects of PAN (Cruz, Juarez‐Nicolas, Tapia, Correa‐Rotter, & Pedraza‐Chaverri, 1994; Kerlin et al., 2012b; Kerlin, Waller, et al., 2015; Waller, Wolfgang, Wiggins, Smoyer, & Kerlin, 2016). Although PAN‐NS is commonly used as a model of minimal change disease (the most common form of idiopathic childhood NS), the mechanisms underlying disease initiation are not identical (Pippin et al., 2009). PAN causes reactive oxygen species‐mediated podocyte DNA damage, whereas the initiating factors of idiopathic childhood NS are ill‐defined and likely multifactorial (i.e. immune, environmental toxin, other) (Berg & Weening, 2004; Dossier et al., 2016; Pippin et al., 2009). Therefore, caution should be exercised in extrapolating these data, despite the similarities between our PAN‐NS and childhood NS data. As expected (Eddy & Symons, 2003), SRNS patients were significantly older than those with SSNS, whereas these groups were otherwise well‐matched. While ETP is known to increase with age, there is no significant change within the childhood range (0.5–17 years) (Haidl, Cimenti, Leschnik, Zach, & Muntean, 2006). In addition, analysis of our current ETP data with the post‐pubertal (>11 years) subjects excluded did not change the overall results (data not shown). Current evidence suggests that platelets may play a role in VTE pathogenesis and it is well‐known that NS may be associated with thrombocytosis (Kerlin et al., 2012a; Wolberg et al., 2015). Meanwhile there are conflicting data regarding the effects of NS on platelet function, which has previously been reported to be both enhanced and impaired during NS (Eneman et al., 2017; Eneman, Levtchenko, van den Heuvel, Van Geet, & Freson, 2016; Svetlov, Moskaleva, Pinelis, Daikhin, & Serebruany, 1999). Our plasma hypercoagulopathy data omit potential contributions from the platelet compartment, although our previously reported whole blood rotational thromboelastometry data correlate well with ETP, suggesting that methods incorporating cellular blood components do not enhance discrimination of hypercoagulopathy (Kerlin, Waller, et al., 2015). Nonetheless, these nuclear receptor agonists may alter megakaryopoiesis and platelet production/function in important ways deserving of further investigation. Similarly, the other components of Virchow's Triad (blood flow and endothelial function) should be considered in future studies.

Both pioglitazone and methylprednisolone simultaneously improved proteinuria and NS‐associated hypercoagulopathy. Pioglitazone enabled a steroid‐sparing treatment strategy in the PAN‐NS rat model. These data are the first to show that NS‐associated hypercoagulopathy improves in concert with therapeutic response. Moreover because the hypercoagulopathy is proportional to disease activity, even a partial disease response (i.e., partial NS remission) may thus reduce hypercoagulopathy and diminish clinical VTE‐risk. In contrast, children with steroid‐resistant NS appear to be persistently hypercoagulopathic and may thus benefit from prolonged anticoagulation therapy, consistent with current treatment guidelines (Monagle et al., 2018). Future studies linking disease activity, hypercoagulopathy, and thrombotic events are needed to realize the full potential of these observations to guide the safe and effective use of anticoagulation for patients with NS. Because these data suggest that any treatment modality that effectively reduces NS disease activity may ameliorate NS‐associated hypercoagulopathy, global hemostatic assays able to detect hypercoagulopathy (e.g., ETP) should be included as valuable secondary outcome variables in future NS clinical trials that employ composite outcomes.

## CONFLICT OF INTEREST

The authors declare no competing financial interests.

## AUTHOR CONTRIBUTIONS

APW, KJW, JK, and MAC conducted experiments, analyzed data, prepared figures, and wrote the manuscript. SA analyzed data, prepared figures, and edited the paper. WES and BAK provided animals and reagents, edited the paper, and were responsible for overseeing and coordinating the study. The PNRC authors (Acknowledgments) enrolled, treated, and contributed the patient data without which this study would have been impossible.

## References

[phy214515-bib-0001] Adeyemo, A. , Esezobor, C. , Solarin, A. , Abeyagunawardena, A. , Kari, J. A. , El Desoky, S. , … Gbadegesin, R. (2018). HLA‐DQA1 and APOL1 as Risk Loci for Childhood‐Onset Steroid‐Sensitive and Steroid‐Resistant Nephrotic Syndrome. American Journal of Kidney Diseases, 71, 399–406. 10.1053/j.ajkd.2017.10.013 29277510 PMC5828864

[phy214515-bib-0002] Agrawal, S. , Chanley, M. A. , Westbrook, D. , Nie, X. , Kitao, T. , Guess, A. J. , … Smoyer, W. E. (2016). Pioglitazone Enhances the Beneficial Effects of Glucocorticoids in Experimental Nephrotic Syndrome. Scientific Reports, 6, 24392 10.1038/srep24392 27142691 PMC4855145

[phy214515-bib-0003] Agrawal, S. , Guess, A. J. , Benndorf, R. , & Smoyer, W. E. (2011). Comparison of direct action of thiazolidinediones and glucocorticoids on renal podocytes: Protection from injury and molecular effects. Molecular Pharmacology, 80, 389–399. 21636793 10.1124/mol.111.071654 PMC3164328

[phy214515-bib-0004] Agrawal, S. , Merchant, M. L. , Kino, J. , Li, M. , Wilkey, D. W. , Gaweda, A. E. , … Smoyer, W. E. (2020). Predicting and defining steroid resistance in pediatric nephrotic syndrome using plasma proteomics. Kidney International Reports, 5, 66–80. 10.1016/j.ekir.2019.09.009 31922062 PMC6943770

[phy214515-bib-0005] Al‐Majed, A. , Bakheit, A. H. , Abdel Aziz, H. A. , Alharbi, H. , & Al‐Jenoobi, F. I. (2016). Pioglitazone. Profiles Drug Subst Excip Relat Methodol, 41, 379–438. 26940171 10.1016/bs.podrm.2015.11.002

[phy214515-bib-0006] Al‐Mugeiren, M. M. , Abdel Gader, A. G. , Al‐Rasheed, S. A. , & Al‐Salloum, A. A. (2006). Tissue factor pathway inhibitor in childhood nephrotic syndrome. Pediatric Nephrology(Berlin, Germany), 21, 771–777. 10.1007/s00467-006-0061-2 16575589

[phy214515-bib-0007] Asharam, K. , Bhimma, R. , David, V. A. , Coovadia, H. M. , Qulu, W. P. , Naicker, T. , … Winkler, C. A. (2018). NPHS2 v260e is a frequent cause of steroid‐resistant nephrotic syndrome in black south african children. Kidney International Reports, 3, 1354–1362. 10.1016/j.ekir.2018.07.017 30450462 PMC6224675

[phy214515-bib-0008] Ay, C. , Dunkler, D. , Simanek, R. , Thaler, J. , Koder, S. , Marosi, C. , … Pabinger, I. (2011). Prediction of venous thromboembolism in patients with cancer by measuring thrombin generation: Results from the Vienna Cancer and Thrombosis Study. Journal of Clinical Oncology : Official Journal of the American Society of Clinical Oncology, 29, 2099–2103. 21464402 10.1200/JCO.2010.32.8294

[phy214515-bib-0009] Banaszak, B. , & Banaszak, P. (2012). The increasing incidence of initial steroid resistance in childhood nephrotic syndrome. Pediatric Nephrology(Berlin, Germany), 27, 927–932. 10.1007/s00467-011-2083-7 PMC3337414 22231438

[phy214515-bib-0010] Besser, M. , Baglin, C. , Luddington, R. , Vlieg, A. V. , & Baglin, T. (2008). High rate of unprovoked recurrent venous thrombosis is associated with high thrombin‐generating potential in a prospective cohort study. Journal of Thrombosis and Haemostasis, 6, 1720–1725. 10.1111/j.1538-7836.2008.03117.x 18680535

[phy214515-bib-0011] Bodary, P. , Vargas, F. , King, S. , Jongeward, K. , Wickenheiser, K. , & Eitzman, D. (2005). IN FOCUS: Pioglitazone protects against thrombosis in a mouse model of obesity and insulin resistance. Journal of Thrombosis and Haemostasis, 3, 2149–2153. 16194192 10.1111/j.1538-7836.2005.01551.x

[phy214515-bib-0012] Brummel‐Ziedins, K. E. , & Wolberg, A. S. (2014). Global assays of hemostasis. Current Opinion in Hematology, 21, 395–403. 10.1097/MOH.0000000000000074 25054908 PMC4163940

[phy214515-bib-0013] Cheng, D. , Gao, H. , & Li, W. (2018). Long‐term risk of rosiglitazone on cardiovascular events ‐ a systematic review and meta‐analysis. Endokrynologia Polska, 69, 381–394. 29952413 10.5603/EP.a2018.0036

[phy214515-bib-0014] Christiansen, C. F. , Schmidt, M. , Lamberg, A. L. , Horvath‐Puho, E. , Baron, J. A. , Jespersen, B. , & Sorensen, H. T. (2014). Kidney disease and risk of venous thromboembolism: A nationwide population‐based case‐control study. Journal of Thrombosis and Haemostasis, 12, 1449–1454. 25040558 10.1111/jth.12652

[phy214515-bib-0015] Citak, A. , Emre, S. , Sirin, A. , Bilge, I. , & Nayir, A. (2000). Hemostatic problems and thromboembolic complications in nephrotic children. Pediatric Nephrology, 14, 138–142. 10.1007/s004670050029 10684364

[phy214515-bib-0016] Cruz, C. , Juarez‐Nicolas, F. , Tapia, E. , Correa‐Rotter, R. , & Pedraza‐Chaverri, J. (1994). Abnormalities of coagulation in experimental nephrotic syndrome. Nephron, 68, 489–496. 10.1159/000188312 7532794

[phy214515-bib-0017] Dargaud, Y. , Wolberg, A. S. , Luddington, R. , Regnault, V. , Spronk, H. , Baglin, T. , … Negrier, C. (2012). Evaluation of a standardized protocol for thrombin generation measurement using the calibrated automated thrombogram: An international multicentre study. Thrombosis Research, 130, 929–934. 22909826 10.1016/j.thromres.2012.07.017

[phy214515-bib-0018] Derebail, V. K. , Rheault, M. N. , & Kerlin, B. A. (2019). Role of direct oral anticoagulants in patients with kidney disease. Kidney International. 10.1016/j.kint.2019.11.027 PMC7093256 32107019

[phy214515-bib-0019] Dossier, C. , Lapidus, N. , Bayer, F. , Sellier‐Leclerc, A. L. , Boyer, O. , de Pontual, L. , … Deschênes, G. (2016). Epidemiology of idiopathic nephrotic syndrome in children: Endemic or epidemic? Pediatric Nephrology(Berlin, Germany), 31, 2299–2308. 10.1007/s00467-016-3509-z 27778092

[phy214515-bib-0020] Eckardt, K. U. , & Kasiske, B. L. (2012). KDIGO clinical practice guideline for glomerulonephritis foreword. Kidney International Supplements, 2, 140–140. 10.1038/kisup.2012.10 25018924 PMC4089711

[phy214515-bib-0021] Eddy, A. A. , & Symons, J. M. (2003). Nephrotic syndrome in childhood. Lancet, 362, 629–639. 10.1016/S0140-6736(03)14184-0 12944064

[phy214515-bib-0022] Eichinger, S. , Hron, G. , Kollars, M. , & Kyrle, P. A. (2008). Prediction of recurrent venous thromboembolism by endogenous thrombin potential and D‐dimer. Clinical Chemistry, 54, 2042–2048. 10.1373/clinchem.2008.112243 18948369

[phy214515-bib-0023] Emani, S. , Zurakowski, D. , Baird, C. W. , Pigula, F. A. , Trenor, C. 3rd , & Emani, S. M. (2013). Hypercoagulability markers predict thrombosis in single ventricle neonates undergoing cardiac surgery. The Annals of Thoracic Surgery, 96, 651–656. 23809731 10.1016/j.athoracsur.2013.04.061

[phy214515-bib-0024] Emani, S. , Zurakowski, D. , Baird, C. W. , Pigula, F. A. , Trenor, C. 3rd , & Emani, S. M. (2014). Hypercoagulability panel testing predicts thrombosis in neonates undergoing cardiac surgery. American Journal of Hematology, 89, 151–155. 10.1002/ajh.23607 24123221

[phy214515-bib-0025] Eneman, B. , Elmonem, M. A. , van den Heuvel, L. P. , Khodaparast, L. , Khodaparast, L. , van Geet, C. , … Levtchenko, E. (2017). Pituitary adenylate cyclase‐activating polypeptide (PACAP) in zebrafish models of nephrotic syndrome. PLoS One, 12, e0182100 10.1371/journal.pone.0182100 28759637 PMC5536324

[phy214515-bib-0026] Eneman, B. , Levtchenko, E. , van den Heuvel, B. , Van Geet, C. , & Freson, K. (2016). Platelet abnormalities in nephrotic syndrome. Pediatric Nephrology(Berlin, Germany), 31, 1267–1279. 10.1007/s00467-015-3173-8 26267676

[phy214515-bib-0027] Erdmann, E. , Dormandy, J. A. , Charbonnel, B. , Massi‐Benedetti, M. , Moules, I. K. , Skene, A. M. , & Investigators, P. R. (2007). The effect of pioglitazone on recurrent myocardial infarction in 2,445 patients with type 2 diabetes and previous myocardial infarction: Results from the PROactive (PROactive 05) Study. Journal of the American College of Cardiology, 49, 1772–1780. 17466227 10.1016/j.jacc.2006.12.048

[phy214515-bib-0028] Glassock, R. J. (2007). Prophylactic anticoagulation in nephrotic syndrome: A clinical conundrum. Journal of the American Society of Nephrology, 18, 2221–2225. 17599972 10.1681/ASN.2006111300

[phy214515-bib-0029] Gooding, J. R. , Agrawal, S. , McRitchie, S. , Acuff, Z. , Merchant, M. L. , Klein, J. B. , … Sumner, S. J. (2020). Predicting and defining steroid resistance in pediatric nephrotic syndrome using plasma metabolomics. Kidney International Reports, 5, 81–93. 10.1016/j.ekir.2019.09.010 31922063 PMC6943762

[phy214515-bib-0030] Govender, M. A. , Fabian, J. , Gottlich, E. , Levy, C. , Moonsamy, G. , Maher, H. , … Ramsay, M. (2019). The podocin V260E mutation predicts steroid resistant nephrotic syndrome in black South African children with focal segmental glomerulosclerosis. Communications Biology, 2, 416 10.1038/s42003-019-0658-1 31754646 PMC6858321

[phy214515-bib-0031] Greenbaum, L. A. , Benndorf, R. , & Smoyer, W. E. (2012). Childhood nephrotic syndrome–current and future therapies. Nature Reviews Nephrology, 8, 445–458. 10.1038/nrneph.2012.115 22688744

[phy214515-bib-0032] Gribouval, O. , Boyer, O. , Hummel, A. , Dantal, J. , Martinez, F. , Sberro‐Soussan, R. , … Servais, A. (2018). Identification of genetic causes for sporadic steroid‐resistant nephrotic syndrome in adults. Kidney International, 94, 1013–1022. 10.1016/j.kint.2018.07.024 30348286

[phy214515-bib-0033] Gribouval, O. , Boyer, O. , Knebelmann, B. , Karras, A. , Dantal, J. , Fourrage, C. , … Servais, A. (2019). APOL1 risk genotype in European steroid‐resistant nephrotic syndrome and/or focal segmental glomerulosclerosis patients of different African ancestries. Nephrology, Dialysis, Transplantation, 34, 1885–1893. 10.1093/ndt/gfy176 29992269

[phy214515-bib-0034] Haidl, H. , Cimenti, C. , Leschnik, B. , Zach, D. , & Muntean, W. (2006). Age‐dependency of thrombin generation measured by means of calibrated automated thrombography (CAT). Thrombosis and Haemostasis, 95, 772–775. 10.1160/TH05-10-0685 16676066

[phy214515-bib-0035] Hodson, E. M. , & Craig, J. C. (2008). Therapies for steroid‐resistant nephrotic syndrome. Pediatric Nephrology(Berlin, Germany), 23, 1391–1394. 10.1007/s00467-008-0792-3 18368428

[phy214515-bib-0036] Hron, G. , Kollars, M. , Binder, B. R. , Eichinger, S. , & Kyrle, P. A. (2006). Identification of patients at low risk for recurrent venous thromboembolism by measuring thrombin generation. JAMA, 296, 397–402. 10.1001/jama.296.4.397 16868297

[phy214515-bib-0037] Isidori, A. M. , Minnetti, M. , Sbardella, E. , Graziadio, C. , & Grossman, A. B. (2015). Mechanisms in endocrinology: The spectrum of haemostatic abnormalities in glucocorticoid excess and defect. European Journal of Endocrinology, 173, R101–113. 25987566 10.1530/EJE-15-0308

[phy214515-bib-0038] Jearath, V. , Vashisht, R. , Rustagi, V. , Raina, S. , & Sharma, R. (2016). Pioglitazone‐induced congestive heart failure and pulmonary edema in a patient with preserved ejection fraction. Journal of Pharmacology & Pharmacotherapeutics, 7, 41–43. 10.4103/0976-500X.179363 27127397 PMC4831491

[phy214515-bib-0039] Jin, H. , Gebska, M. A. , Blokhin, I. O. , Wilson, K. M. , Ketsawatsomkron, P. , Chauhan, A. K. , … Lentz, S. R. (2015). Endothelial PPAR‐gamma protects against vascular thrombosis by downregulating P‐selectin expression. Arteriosclerosis, Thrombosis, and Vascular Biology, 35, 838–844. 10.1161/ATVBAHA.115.305378 PMC4376629 25675995

[phy214515-bib-0040] Johannesdottir, S. A. , Horváth‐Puhó, E. , Dekkers, O. M. , Cannegieter, S. C. , Jørgensen, J. O. L. , Ehrenstein, V. , … Sørensen, H. T. (2013). Use of glucocorticoids and risk of venous thromboembolism: A nationwide population‐based case‐control study. JAMA Internal Medicine, 173, 743–752. 23546607 10.1001/jamainternmed.2013.122

[phy214515-bib-0041] KDIGO Clinical Practice Guideline for Glomerulonephritis . (2012). Kidney International Supplement, 2, 139–274.

[phy214515-bib-0042] Kelddal, S. , Nykjaer, K. M. , Gregersen, J. W. , & Birn, H. (2019). Prophylactic anticoagulation in nephrotic syndrome prevents thromboembolic complications. BMC Nephrology, 20, 139 10.1186/s12882-019-1336-8 31023275 PMC6482554

[phy214515-bib-0043] Kerlin, B. A. , Ayoob, R. , & Smoyer, W. E. (2012). Epidemiology and pathophysiology of nephrotic syndrome–associated thromboembolic disease. Clinical Journal of the American Society of Nephrology, 7, 513–520. 10.2215/CJN.10131011 22344511 PMC3302669

[phy214515-bib-0044] Kerlin, B. A. , Ayoob, R. , & Smoyer, W. E. (2012). Epidemiology and pathophysiology of nephrotic syndrome‐associated thromboembolic disease. Clinical Journal of the American Society of Nephrology : CJASN, 7, 513–520. 10.2215/CJN.10131011 22344511 PMC3302669

[phy214515-bib-0045] Kerlin, B. A. , Blatt, N. B. , Fuh, B. , Zhao, S. , Lehman, A. , Blanchong, C. , Mahan, J. D. , … Smoyer, W. E. (2009). Epidemiology and risk factors for thromboembolic complications of childhood nephrotic syndrome: A Midwest Pediatric Nephrology Consortium (MWPNC) study. The Journal of Pediatrics, 110(e101), 105–110. 10.1016/j.jpeds.2009.01.070 PMC3685482 19394032

[phy214515-bib-0046] Kerlin, B. A. , Smoyer, W. E. , Tsai, J. , & Boulet, S. L. (2015). Healthcare burden of venous thromboembolism in childhood chronic renal diseases. Pediatric Nephrology(Berlin, Germany), 30, 829–837. 10.1007/s00467-014-3008-z PMC4375065 25487668

[phy214515-bib-0047] Kerlin, B. A. , Waller, A. P. , Sharma, R. , Chanley, M. A. , Nieman, M. T. , & Smoyer, W. E. (2015). Disease severity correlates with thrombotic capacity in experimental nephrotic syndrome. Journal of the American Society of Nephrology, 26, 3009–3019. 10.1681/ASN.2014111097 25855774 PMC4657841

[phy214515-bib-0048] Khan, S. , Khan, S. , Imran, M. , Pillai, K. K. , Akhtar, M. , & Najmi, A. K. (2013). Effects of pioglitazone and vildagliptin on coagulation cascade in diabetes mellitus–targeting thrombogenesis. Expert Opinion on Therapeutic Targets, 17, 627–639. 10.1517/14728222.2013.764991 23356568

[phy214515-bib-0049] Lee, T. , Biddle, A. K. , Lionaki, S. , Derebail, V. K. , Barbour, S. J. , Tannous, S. , … Nachman, P. H. (2014). Personalized prophylactic anticoagulation decision analysis in patients with membranous nephropathy. Kidney International, 85, 1412–1420. 10.1038/ki.2013.476 24336031 PMC4040154

[phy214515-bib-0050] Li, D. , Chen, K. , Sinha, N. , Zhang, X. , Wang, Y. , Sinha, A. K. , … Mehta, J. L. (2005). The effects of PPAR‐gamma ligand pioglitazone on platelet aggregation and arterial thrombus formation. Cardiovascular Research, 65, 907–912. 15721871 10.1016/j.cardiores.2004.11.027

[phy214515-bib-0051] Lilova, M. I. , Velkovski, I. G. , & Topalov, I. B. (2000). Thromboembolic complications in children with nephrotic syndrome in Bulgaria (1974–1996). Pediatric Nephrology(Berlin, Germany), 15, 74–78. 10.1007/s004679900253 11095017

[phy214515-bib-0052] Lionaki, S. , Derebail, V. K. , Hogan, S. L. , Barbour, S. , Lee, T. , Hladunewich, M. , … Reich, H. N. (2012). Venous thromboembolism in patients with membranous nephropathy. Clinical Journal of the American Society of Nephrology, 7, 43–51. 10.2215/CJN.04250511 22076873 PMC3265338

[phy214515-bib-0053] Longui, C. A. (2007). Glucocorticoid therapy: Minimizing side effects. Jornal De Pediatria, 83, S163–177. 18000630 10.2223/JPED.1713

[phy214515-bib-0054] Loscalzo, J. (2013). Venous thrombosis in the nephrotic syndrome. The New England Journal of Medicine, 368, 956–958. 10.1056/NEJMcibr1209459 23465106

[phy214515-bib-0055] Mahmoodi, B. , ten Kate, M. , & Waanders, F. (2008). High absolute risk and predictors of venous and arterial thromboembolic events in patients with nephrotic syndrome: Results from a large retrospective cohort study. Journal of Vascular Surgery, 48, 1633. 10.1161/CIRCULATIONAHA.107.716951 18158362

[phy214515-bib-0056] Majoor, C. J. , Sneeboer, M. M. , de Kievit, A. , Meijers, J. C. , van der Poll, T. , Lutter, R. , … Kamphuisen, P. W. (2016). The influence of corticosteroids on hemostasis in healthy subjects. Journal of Thrombosis and Haemostasis, 14, 716–723. 10.1111/jth.13265 26791678

[phy214515-bib-0057] Mehls, O. , Andrassy, K. , Koderisch, J. , Herzog, U. , & Ritz, E. (1987). Hemostasis and thromboembolism in children with nephrotic syndrome: Differences from adults. Journal of Pediatrics, 110, 862–867. 3585601 10.1016/s0022-3476(87)80397-9

[phy214515-bib-0058] Miyawaki, Y. , Suzuki, A. , Fujita, J. , Maki, A. , Okuyama, E. , Murata, M. , … Kojima, T. (2012). Thrombosis from a prothrombin mutation conveying antithrombin resistance. New England Journal of Medicine, 366, 2390–2396. 10.1056/NEJMoa1201994 22716977

[phy214515-bib-0059] Monagle, P. , Cuello, C. A. , Augustine, C. , Bonduel, M. , Brandao, L. R. , Capman, T. , … Vesely, S. K. (2018). American Society of Hematology 2018 Guidelines for management of venous thromboembolism: Treatment of pediatric venous thromboembolism. Blood Advance, 2, 3292–3316. 10.1182/bloodadvances.2018024786 PMC6258911 30482766

[phy214515-bib-0060] Ødegård, O. (1975). Evaluation of an amidolytic heparin cofactor assay method. Thrombosis Research, 7, 351–360. 10.1016/0049-3848(75)90193-0 51519

[phy214515-bib-0061] Ozsoylu, S. , Strauss, H. S. , & Diamond, L. K. (1962). Effects of corticosteroids on coagulation of the blood. Nature, 195, 1214–1215. 10.1038/1951214a0 14038141

[phy214515-bib-0062] Park, S. J. , & Shin, J. I. (2011). Complications of nephrotic syndrome. Korean Journal of Pediatrics, 54, 322–328. 10.3345/kjp.2011.54.8.322 22087198 PMC3212701

[phy214515-bib-0063] Pelham, C. J. , Keen, H. L. , Lentz, S. R. , & Sigmund, C. D. (2013). Dominant negative PPARgamma promotes atherosclerosis, vascular dysfunction, and hypertension through distinct effects in endothelium and vascular muscle. American Journal of Physiology Regulatory, Integrative and Comparative Physiology, 304, R690–701. 10.1152/ajpregu.00607.2012 PMC3652079 23447133

[phy214515-bib-0064] Pfutzner, A. , Marx, N. , Lubben, G. , Langenfeld, M. , Walcher, D. , Konrad, T. , & Forst, T. (2005). Improvement of cardiovascular risk markers by pioglitazone is independent from glycemic control ‐ Results from the pioneer study. Journal of the American College of Cardiology, 45, 1925–1931. 10.1016/j.jacc.2005.03.041 15963388

[phy214515-bib-0065] Picard, V. , Ersdal‐Badju, E. , & Bock, S. C. (1995). Partial glycosylation of antithrombin III asparagine‐135 is caused by the serine in the third position of its N‐glycosylation consensus sequence and is responsible for production of the beta‐antithrombin III isoform with enhanced heparin affinity. Biochemistry, 34, 8433–8440. 10.1021/bi00026a026 7599134

[phy214515-bib-0066] Pippin, J. W. , Brinkkoetter, P. T. , Cormack‐Aboud, F. C. , Durvasula, R. V. , Hauser, P. V. , Kowalewska, J. , … Shankland, S. J. (2009). Inducible rodent models of acquired podocyte diseases. American Journal of Physiology. Renal Physiology, 296, F213–229. 10.1152/ajprenal.90421.2008 18784259

[phy214515-bib-0067] Ransom, R. F. , Lam, N. G. , Hallett, M. A. , Atkinson, S. J. , & Smoyer, W. E. (2005). Glucocorticoids protect and enhance recovery of cultured murine podocytes via actin filament stabilization. Kidney International, 68, 2473–2483. 10.1111/j.1523-1755.2005.00723.x 16316324

[phy214515-bib-0068] Ransom, R. F. , Vega‐Warner, V. , Smoyer, W. E. , & Klein, J. (2005). Differential proteomic analysis of proteins induced by glucocorticoids in cultured murine podocytes. Kidney International, 67, 1275–1285. 10.1111/j.1523-1755.2005.00205.x 15780080

[phy214515-bib-0069] Rose, L. J. , Dunn, M. E. , Allegret, V. , & Bédard, C. (2011). Effect of prednisone administration on coagulation variables in healthy B eagle dogs. Veterinary Clinical Pathology, 40, 426–434. 10.1111/j.1939-165X.2011.00364.x 22093028

[phy214515-bib-0070] Schlegel, N. (1997). Thromboembolic risks and complications in nephrotic children. Seminars in Thrombosis and Hemostasis, 23, 271–280. 10.1055/s-2007-996100 9255908

[phy214515-bib-0071] Schonenberger, E. , Ehrich, J. H. , Haller, H. , & Schiffer, M. (2011). The podocyte as a direct target of immunosuppressive agents. Nephrology, Dialysis, Transplantation : Official Publication of the European Dialysis and Transplant Association ‐ European Renal Association, 26, 18–24. 10.1093/ndt/gfq617 20937691

[phy214515-bib-0072] Singhal, R. , & Brimble, K. S. (2006). Thromboembolic complications in the nephrotic syndrome: Pathophysiology and clinical management. Thrombosis Research, 118, 397–407. 15990160 10.1016/j.thromres.2005.03.030

[phy214515-bib-0074] Smyth, S. , & Jennings, J. (2005). COMMENTARY: PPARγ agonists: A new strategy for antithrombotic therapy. Journal of Thrombosis and Haemostasis, 3, 2147–2148. 16194191 10.1111/j.1538-7836.2005.01585.x

[phy214515-bib-0075] Sonneveld, R. , Hoenderop, J. G. , Isidori, A. M. , Henique, C. , Dijkman, H. B. , Berden, J. H. , … Nijenhuis, T. (2017). Sildenafil Prevents Podocyte Injury via PPAR‐gamma‐Mediated TRPC6 Inhibition. Journal of the American Society of Nephrology, 28, 1491–1505. 27895156 10.1681/ASN.2015080885 PMC5407711

[phy214515-bib-0076] Sonnevi, K. , Tchaikovski, S. N. , Holmstrom, M. , Antovic, J. P. , Bremme, K. , Rosing, J. , & Larfars, G. (2013). Obesity and thrombin‐generation profiles in women with venous thromboembolism. Blood Coagulation & Fibrinolysis, 24, 547–553. 10.1097/MBC.0b013e32835f93d5 23470648

[phy214515-bib-0077] Svetlov, S. I. , Moskaleva, E. S. , Pinelis, V. G. , Daikhin, Y. , & Serebruany, V. L. (1999). Decreased intraplatelet Ca2+ release and ATP secretion in pediatric nephrotic syndrome. Pediatric Nephrology(Berlin, Germany), 13, 205–208. 10.1007/s004670050593 10353406

[phy214515-bib-0078] Thrombosis: a major contributor to the global disease burden . (2014). J Thromb Haemost, 12, 1580–1590. 10.1111/jth.12698 25302663

[phy214515-bib-0079] Trachtman, H. , Vento, S. , Herreshoff, E. , Radeva, M. , Gassman, J. , Stein, D. T. , … Gipson, D. S. (2015). Efficacy of galactose and adalimumab in patients with resistant focal segmental glomerulosclerosis: Report of the font clinical trial group. BMC Nephrology, 16, 111. 26198842 10.1186/s12882-015-0094-5 PMC4511259

[phy214515-bib-0080] Tripodi, A. , Martinelli, I. , Chantarangkul, V. , Battaglioli, T. , Clerici, M. , & Mannucci, P. M. (2007). The endogenous thrombin potential and the risk of venous thromboembolism. Thrombosis Research, 121, 353–359. 10.1016/j.thromres.2007.04.012 17560633

[phy214515-bib-0081] Ueda, N. (1990). Effect of corticosteroids on some hemostatic parameters in children with minimal change nephrotic syndrome. Nephron, 56, 374–378. 10.1159/000186178 2079995

[phy214515-bib-0082] Ueda, N. , Kawaguchi, S. , Niinomi, Y. , Nonoda, T. , Matsumoto, J. , Ohnishi, M. , & Yasaki, T. (1987). Effect of corticosteroids on coagulation factors in children with nephrotic syndrome. Pediatric Nephrology(Berlin, Germany), 1, 286–289. 10.1007/BF00849225 3153290

[phy214515-bib-0083] Ulinski, T. , Guigonis, V. , Baudet‐Bonneville, V. , Auber, F. , Garcette, K. , & Bensman, A. (2003). Mesenteric thrombosis causing short bowel syndrome in nephrotic syndrome. Pediatric Nephrology(Berlin, Germany), 18, 1295–1297. 10.1007/s00467-003-1281-3 14564498

[phy214515-bib-0084] van den Berg, J. G. , & Weening, J. J. (2004). Role of the immune system in the pathogenesis of idiopathic nephrotic syndrome. Clinical Science (Lond), 107, 125–136. 10.1042/CS20040095 15157184

[phy214515-bib-0085] van Hylckama, V. A. , Baglin, C. A. , Luddington, R. , MacDonald, S. , Rosendaal, F. R. , & Baglin, T. P. (2015). The risk of a first and a recurrent venous thrombosis associated with an elevated D‐dimer level and an elevated thrombin potential: Results of the THE‐VTE study. Journal of Thrombosis and Haemostasis, 13, 1642–1652. 26178257 10.1111/jth.13043

[phy214515-bib-0086] van Hylckama, V. A. , Christiansen, S. C. , Luddington, R. , Cannegieter, S. C. , Rosendaal, F. R. , & Baglin, T. P. (2007). Elevated endogenous thrombin potential is associated with an increased risk of a first deep venous thrombosis but not with the risk of recurrence. British Journal of Haematology, 138, 769–774. 10.1111/j.1365-2141.2007.06738.x 17760809

[phy214515-bib-0087] van Zaane, B. , Nur, E. , Squizzato, A. , Gerdes, V. E. , Büller, H. R. , Dekkers, O. M. , & Brandjes, D. P. (2010). Systematic review on the effect of glucocorticoid use on procoagulant, anti‐coagulant and fibrinolytic factors. Journal of Thrombosis and Haemostasis, 8, 2483–2493. 10.1111/j.1538-7836.2010.04034.x 20735729

[phy214515-bib-0088] Waller, A. P. , Wolfgang, K. J. , Wiggins, R. C. , Smoyer, W. E. , & Kerlin, B. A. (2016). Nephrotic syndrome clots are resistant to fibrinolysis despite elevated plasmin generation. Blood, 128, 3767–3767. 10.1182/blood.V128.22.3767.3767

[phy214515-bib-0089] Wilcox, R. , Kupfer, S. , & Erdmann, E. (2008). and investigators PS. Effects of pioglitazone on major adverse cardiovascular events in high‐risk patients with type 2 diabetes: Results from PROspective pioglitAzone Clinical Trial In macro Vascular Events (PROactive 10). American Heart Journal, 155, 712–717. 18371481 10.1016/j.ahj.2007.11.029

[phy214515-bib-0090] Wolberg, A. S. , Rosendaal, F. R. , Weitz, J. I. , Jaffer, I. H. , Agnelli, G. , Baglin, T. , & Mackman, N. (2015). Venous thrombosis. Nature Reviews Disease Primers, 1, 15006 10.1038/nrdp.2015.6 27189130

[phy214515-bib-0091] Yaseen, A. , Tresa, V. , Lanewala, A. A. , Hashmi, S. , Ali, I. , Khatri, S. , & Mubarak, M. (2017). Acute kidney injury in idiopathic nephrotic syndrome of childhood is a major risk factor for the development of chronic kidney disease. Renal Failure, 39, 323–327. 10.1080/0886022X.2016.1277743 28093933 PMC6014292

